# Perceived usability of the National Competence Based Catalogue of Learning Objectives for Undergraduate Medical Education by medical educators at the Hannover Medical School

**DOI:** 10.3205/zma001163

**Published:** 2018-05-15

**Authors:** Sandra Steffens, Volker Paulmann, Jasper Mecklenburg, Konstantin Büttner, Marianne Behrends

**Affiliations:** 1 Hannover Medical School, Dean's Office, Hannover, Germany; 2 Hannover Medical School, Peter L. Reichertz Institute for Medical Informatics, Hannover, Germany

**Keywords:** National Competence Based Catalogue of Learning Objectives for Undergraduate Medical Education (NKLM), usability, teaching, curriculum

## Abstract

**Background: **The aim of this study was to assess usability and identify possible challenges in the implementation of the National Competence Based Catalogue of Learning Objectives for Undergraduate Medical Education (NKLM) among medical educators.

**Methods:** A comprehensive survey among experienced medical educators (responsible for the teaching content and didactical development in their module/field) based on the System Usability Scale (SUS) was carried out focusing on the awareness, usability and challenges of the NKLM.

**Results: **The questionnaire was completed by 52 of the 64 addressed educators. Most of the participants had 6-10 years of teaching experience. 30% of the educators were not familiar with the NKLM. During the evaluation of the NKLM, usability was rather poorly rated. However, 71.9% of medical educators agreed that the various aspects of the medical professions were well integrated in the NKLM with only 12,5% stating that they would not use the NKLM for teaching and lesson preparation.

**Conclusion: **The awareness and promotion of the NKLM need to be improved. Furthermore, these data suggest that – although difficult to use – there is a solid acceptance of the content of the NKLM. Medical educators seem to be willing to use the NKLM. Therefore, further attempts to support colleagues with the handling of the NKLM seem to be inevitable to pave the way for a competency-based curricular change.

## Zusammenfassung

**Hintergrund: **Das Ziel der vorliegenden Studie war es, die Gebrauchstauglichkeit des Nationalen Kompetenzbasierten Lernzielkatalogs Medizin (NKLM) aus der Sicht von Lehrenden im Studienfach Humanmedizin zu bewerten und mögliche Hindernisse bei seiner Einführung zu identifizieren.

**Methodik: **Es wurde eine standardisierte Befragung unter den Lehrverantwortlichen, die für die inhaltliche und didaktische Gestaltung der jeweiligen Module verantwortlich sind, durchgeführt. Basierend auf dem System Usability Scale (SUS) wurden neben der Gebrauchstauglichkeit auch die generelle Vertrautheit sowie die Schwierigkeiten im Umgang mit dem NKLM untersucht.

**Ergebnisse: **Der Fragebogen wurde von 52 der 64 befragten Lehrverantwortlichen ausgefüllt. Die meisten der befragten Personen verfügen über 6-10 Jahre Lehrerfahrung. 30% waren nicht mit dem NKLM vertraut. Die Evaluation verdeutlicht, dass die Gebrauchstauglichkeit des NKLM als unzureichend bewertet wird. Jedoch stimmen 71,9% der Lehrverantwortlichen der Aussage zu, dass die verschiedenen Aspekte des Arztberufes gut im NKLM dargestellt sind. Lediglich 12,5% können sich nicht vorstellen, den NKLM für die Lehre und die Unterrichtsvorbereitung zu nutzen.

**Schlussfolgerung: **Die Wahrnehmung und Verbreitung des NKLM müssen verbessert werden. Des Weiteren legen die Daten nahe, dass – trotz des schwierigen Umgangs mit dem NKLM – die inhaltliche Akzeptanz gegeben ist. Die befragten Lehrverantwortlichen zeigen die Bereitschaft, mit dem NKLM zu arbeiten. Um diese Bereitschaft zu stärken müssen weitere Anstrengungen unternommen werden, um die Lehrenden auf dem Weg zum kompetenzbasierten Curriculum zu unterstützen.

## Introduction

After nearly six years of development and controversial debates, finally in 2015 the National Competence Based Catalogue of Learning Objectives for Undergraduate Medical Education (NKLM) was released (http://www.nklm.de). It defines a wide range of competencies in communication, knowledge, professional skills, clinical judgment, emotions and reflection that enable a medical professional to act effectively and responsibly for the benefit of individual patients and the community [1].

The orientation towards a more competency based medical education in Germany followed reform initiatives that were already introduced in the first decade of the new millennium when new regulations in the German National Guidelines for Medical Education were adopted (https://www.gesetze-im-internet.de/_appro_2002/BJNR240500002.html). Faculties were allowed to establish model educational programs in order to generate new impulses and enable them to keep pace with international developments (https://www.gesetze-im-internet.de/_appro_2002/BJNR240500002.html). 

In 2005, the Hannover Medical School (MHH) introduced the *Hannover integrated adaptive practice-related learning concept *(HannibaL), a model curriculum focusing on professional medical skills and patient centred approaches. Thus – with HannibaL – already elements of the current NKLM-related reform initiative such as patient- and practice-related competencies like communication and practical skills were introduced to undergraduate medical education.

Just recently the “Masterplan 2020” has been released, stating that the NKLM will initially have the status of recommendations for restructuring medical curricula before it will be mandatory for medical education in Germany (https://www.bmbf.de/de/masterplan-medizinstudium-2020-4024.html). Medical faculties were called upon to compare their existing curricula with the NKLM and gather practical experience over the next years. Considering the catalogue’s volume, consisting of 234 competencies, 281 sub-competencies and 1958 learning objectives and its unambiguous subject assignment, this appeal revealed to be challenging [2]. In addition, this is the first time since over 40 years that a nationwide catalogue of learning objectives has been released in Germany [3]. A recently published multicentre study in south of Germany by Lammerding-Koeppel et al. stated that faculty members were not willing to deal with the NKLM emphasizing the need for validated concepts and well researched, motivational strategies are essential [4], [5]. However, it remains unclear why according to the study by Lammerding-Koeppel et al. [4], [5] medical educators seem to be resisting the NKLM and if this is due to its usability.

Therefore, the aim of this study was to assess usability and identify possible challenges in the implementation of the NKLM among medical educators using the framework of a comprehensive questionnaire study. 

## Methods

### Development of the questionnaire

To the best of our knowledge, no validated methods have been published so far that can be used to determine the usability of the NKLM among medical educators. Therefore, a questionnaire, which formed the basis of the data collection in the framework of the presented study, was developed in six focus group sessions, each consisting of three to four participants (three with many years of experience in medical education, two with added experience in development and evaluation of questionnaires in medical education and one with extensive experience regarding quality assurance of medical education). As a suitable instrument, the validated System Usability Scale (SUS) was used as groundwork [6] and a survey developed to evaluate the NKLM with regard to awareness, usability and applicability. Basically, part of the survey consisted of ten items that contribute to a validated test score [7]. A score of 100 represents a perfect usability. Thus, the tool can be used to compare different systems as well as different stages of development [6]. According to Lewis [8] two dimensions are incorporated in the SUS: The factor usability (8 Items) and the factor learnability (2 Items). In order to adopt the questions to the NKLM context, wording and order were slightly changed. This was not conceived to alter the character of subjective evaluations but to define the system adequately. In Item 5 wording (“various functions in this system“) was replaced (“various aspects of the medical professions”) to illustrate the function of the NKLM. “Using the system” (Item 9) was replaced by “identifying the learning objectives of my field in the NKLM”, to point to the character as a learning media. In Item 3 a “technical person” was substituted by “experienced person”. Otherwise, “this system” was consequently replaced by “the NKLM” (see Table 1 (Tab. 1)). Because of the new order of the items learnability is in contrast to the original SUS order represented by items 3 and 10. In order to explore possible boundaries for the NKLM the role of teaching experience and gender of the educators was analysed. Additionally, two free-text open questions regarding the structure and aim of the NKLM were included. It took approximately 5-10 minutes to complete. The survey was conducted as part of the NKLM mapping process following the faculty wide kick-off meeting in September 2016. 

#### Participants 

From September 2016 to February 2017, 62 medical educators who were responsible for the teaching content and didactical development at the Hannover Medical School were questioned. The medical educators were mainly physicians from different medical fields (e.g. surgery, internal medicine, general medicine, gynaecology/obstetrics) and academics from other disciplines (e.g. physics, biochemistry, psychology, sociology, public health) responsible for their curriculum. 

#### Statistical analyses 

The SUS score was calculated according to the procedure described by Lewis and Sauro (8): Each item – from 1 (“strongly disagree”) to 5 (“strongly agree”) – is transformed to a value between 0 to 4. For positively worded items (4, 1, 5, 7 and 9), the score value is the scale position minus 1. For negatively worded items (2, 3, 6, 8 and 10), it is 5 minus the scale position. To calculate the overall SUS score, the sum of the 10 items is multiplied by 2.5. Thus, SUS scores range from 0 to 100.

In addition to the score, single items were analyzed. In order to emphasize the character of a screening tool we report the percentages of participants agreeing or disagreeing with a particular statement. Therefore, the two outer categories on each side of the scale were aggregated e.g., “Strongly Agree and Agree”, into a single value. The student’s t-test was used for mean differences between men and women and teaching experience. Two-sided p-values below 0.05 were considered statistically significant. Usability and learnability were calculated according to the procedure described by Lewis and Sauro [8]. Age and gender were analyzed using descriptive statistics. SPSS 25.0 (USA) was used for statistical assessment.

## Results

From 62 distributed questionnaires 52 were fully completed (response rate 84%). Five questionnaires – where the SUS was filled-in incompletely – were excluded. In one questionnaire teaching experience and gender were missing. Most of the participants had 6-10 years of teaching experience and 20 (39.2%) were female. Table 2 (Tab. 2) shows the characteristics of the survey participants regarding teaching experience and gender. 15 (30%) educators were not familiar with the NKLM and, therefore, could not answer the 10 items of the NKLM SUS. 

**Figure 1:**
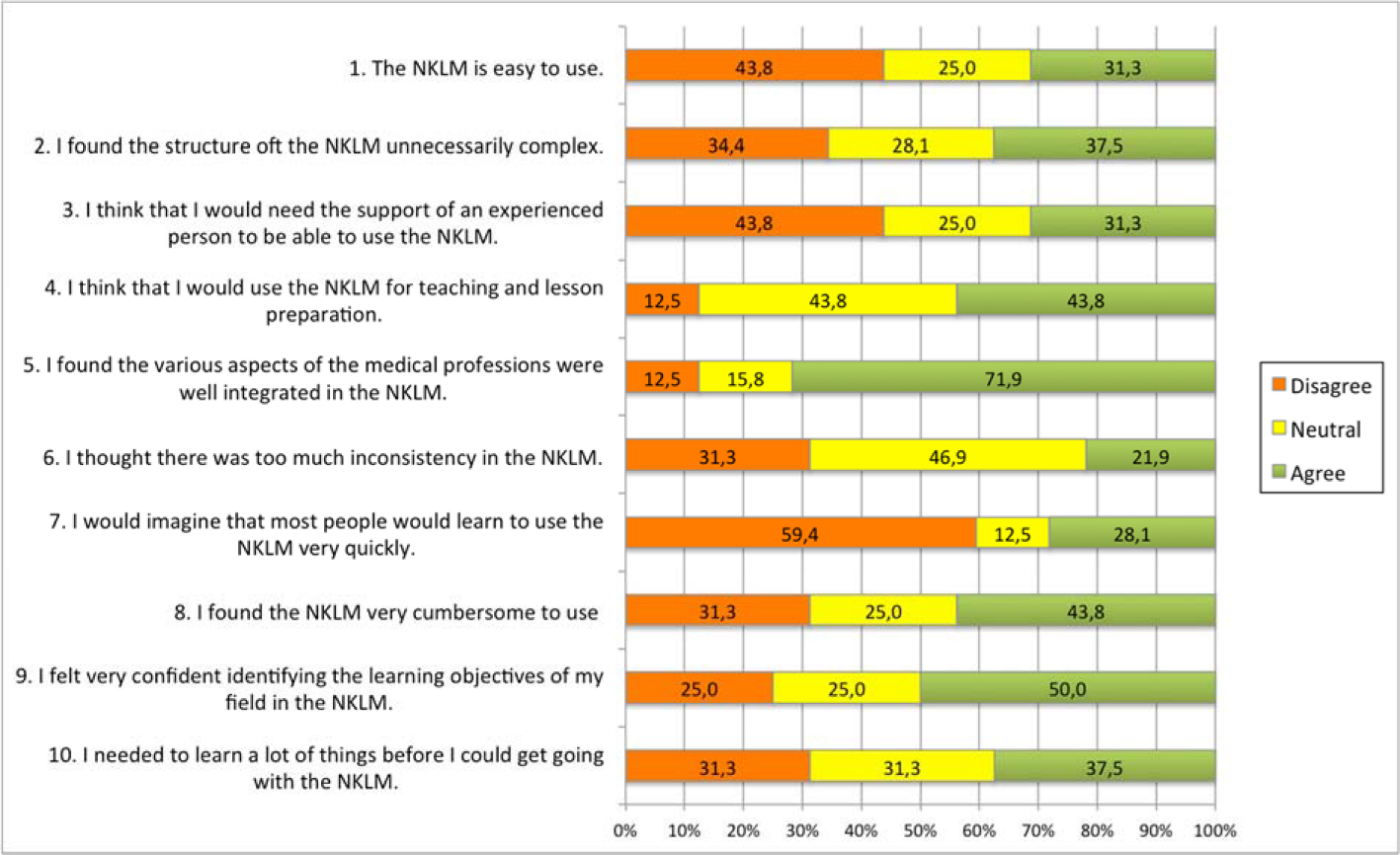
Responses to individual statements in the NKLM System Usability Scale.

32 medical educators completed the System Usability Scale. The average SUS score was 52.7 (standard deviation of 17,7), ranging from 27.5 to 87.5. Interestingly, 71.9% of medical educators agreed that the various aspects of the medical professions were well integrated in the NKLM (Item 5). However, 43.2% of medical educators disagreed with the statement that the NKLM is easy to use with 45.9% agreeing with the statement that the NKLM is very cumbersome to use (Items 1 and 8). In addition, 31.1% agreed to need the support of an experienced person to be able to use the NKLM (Item 3). In contrast, 45.9% think that they would use the NKLM for teaching and lesson preparation (Item 4). 37.5% found the structure of the NKLM unnecessarily complex (Item 2), 59.4% could not imagine that most people would learn to use the NKLM very quickly (Item 7) and 37.5% believe that one needs to learn a lot of things before getting along with the NKLM (Item 10). Even though, 21.9 % thought there were too many inconsistencies in the NKLM (Item 6), 50% agreed with the statement that they felt very confident identifying the learning objectives of their field in the NKLM (Item 9). Table 1 (Tab. 1) and figure 1 (Fig. 1) display the mean responses to individual NKLM SUS statements. 

With regard to gender and teaching experience, there are only slight differences in the SUS score that did not reach statistical significance. 

## Discussion

Any foreshadowing of even the slightest bit of change can already cause distress among individuals [9]. Doubled with the well-known fact that educational institutions are characterized by a rather traditional culture that strives to resist any changes, the implementation of the NKLM is not only novel but could also be considered a bold move [9], [10]. A just recently published multicentre study of medical faculties in south of Germany stated that faculty members were not willing to deal with the NKLM emphasizing the need for validated concepts and well-researched, motivational strategies are essential [4], [5]. Therefore, as first step in the implementation of the NKLM in the Hannover Medical School, the awareness and usability were examined using the framework of a comprehensive questionnaire study. This approach fits best to step one “needs and problem identification” within the framework of the curriculum development in medical education by Thomas et al. [11]. 

In our study almost 30% of the surveyed teachers indicated in response to the first question of the questionnaire that they were not familiar with the NKLM. Considering the fact that in our sample mainly experts in medical education were included, the number of educators who are not familiar enough with the NKLM to make a reasonable judgement is high. Therefore, the promotion of the NKLM needs to be improved. On the one hand, the benefit of a new curriculum needs to be communicated in a wider range. This process involves political and administrative institutions (e.g. Ministries of Education/Science) as well as vocational medical representations (e.g. Medical Association, Professional Society, National Association of Statutory Health Insurance Physicians). Frank and Danoff [12] identified several elements that have contributed to a successful “social marketing” strategy when the CanMeds framework was implemented in Canada and abroad. It includes frequent newsletters, networking with highly motivated educators (“champions”) and the development of a pool of well-informed spokespersons [12]. Therefore, as a key element of the implementation strategy in our faculty, a medical educator was appointed as NKLM instructor. On the other hand, on faculty level, the pervasion should be deepened by addressing informal (e.g. teaching networks) as well as formal channels (e.g. academic planning committee, senate). Bland et al. [13] pointed to the fact that at medical schools face-to-face interaction is helpful to convey a change agenda. In addition, to support the new initiative rewards for early adopters and innovative projects could also be helpful [13].

It is well known from research on usability and technology acceptance that the willingness to use new products or innovations can depend on the perceived usefulness, the learnability and the intention to use [14], [15]. According to the ISO-definition 9241-11, “Usability is the extent to which a product can be used by specified users to achieve specified goals with effectiveness, efficiency, and satisfaction in a specified context of use.” [16]. Learnability describes how effective usability can be learned. Often, usability testing is conducted in the framework of software engineering. Yet, other products and services may well be analysed from a usability point of view. It therefore seems sensible to take these aspects into consideration with regard to the acceptance of the NKLM. The usability aspects of the NKLM remain the biggest challenge in terms of change management. A majority in our sample finds it not easy to use (item 8) – a result that is not surprising considering the mere extent of learning objectives. 

Interestingly, even though there is scepticism regarding the usability of the “technical side” of NKLM (items 1,2, 6 and 8) among medical educators, the overall approach of the NKLM (item 5: *I found the various aspects of the medical professions were well integrated in the NKLM*) is appreciated. 72% found, that “the various aspects of the medical professions were well integrated in the NKLM” (item 5). According to Frank and Danoff [12] the acceptance of the content is one of the most important requirements when introducing a new competency based framework. In addition, only a minority finds it difficult to identify subject-related learning objectives in the NKLM with just 13% stating that they would not use the NKLM for teaching preparing their teaching lessons (item 4). 

To the best of our knowledge, there is only little empirical evidence for the influence of individual-related factors on the introduction of learning catalogues. Reflections of successful realizations of other learning catalogues – as the CanMEDS initiative or the Swiss catalogue of learning objectives (SCLO) – rather focus on organizational aspects [12], the acceptance of the content [17] or the development of the catalogue itself [18]. From a cultural point of view, academia in general [19] and academic medicine [20] have been described differently by men and women with regard to participation, chances, job advancement and perception by students and colleagues. In a study by O’Sullivan teaching experience and gender played a role among Irish dental faculty members regarding their interest in faculty development. However, in contrast to O’Sullivan [21], in our sample there were no significant differences among educators with regard to gender, age or teaching experience. There are some limitations to our study, e.g. sample size and single-centre approach. 

In conclusion, as there are still experienced medical educators that were not familiar with the NKLM, the awareness and promotion of the NKLM need to be improved as well as the benefits. Furthermore, these data suggest that, although difficult to use, there is a solid acceptance of the content of the NKLM. Medical educators seem to be willing to use the NKLM. Therefore, further attempts aiming to improve the usability as well as supporting colleagues with the handling of the NKLM seem to be inevitable to pave the way for a competency-based curricular change.

## Authors

Sandra Steffens and Volker Paulmann have contributed equally to this work.

## Competing interests

The authors declare that they have no competing interests. 

## Figures and Tables

**Table 1:**
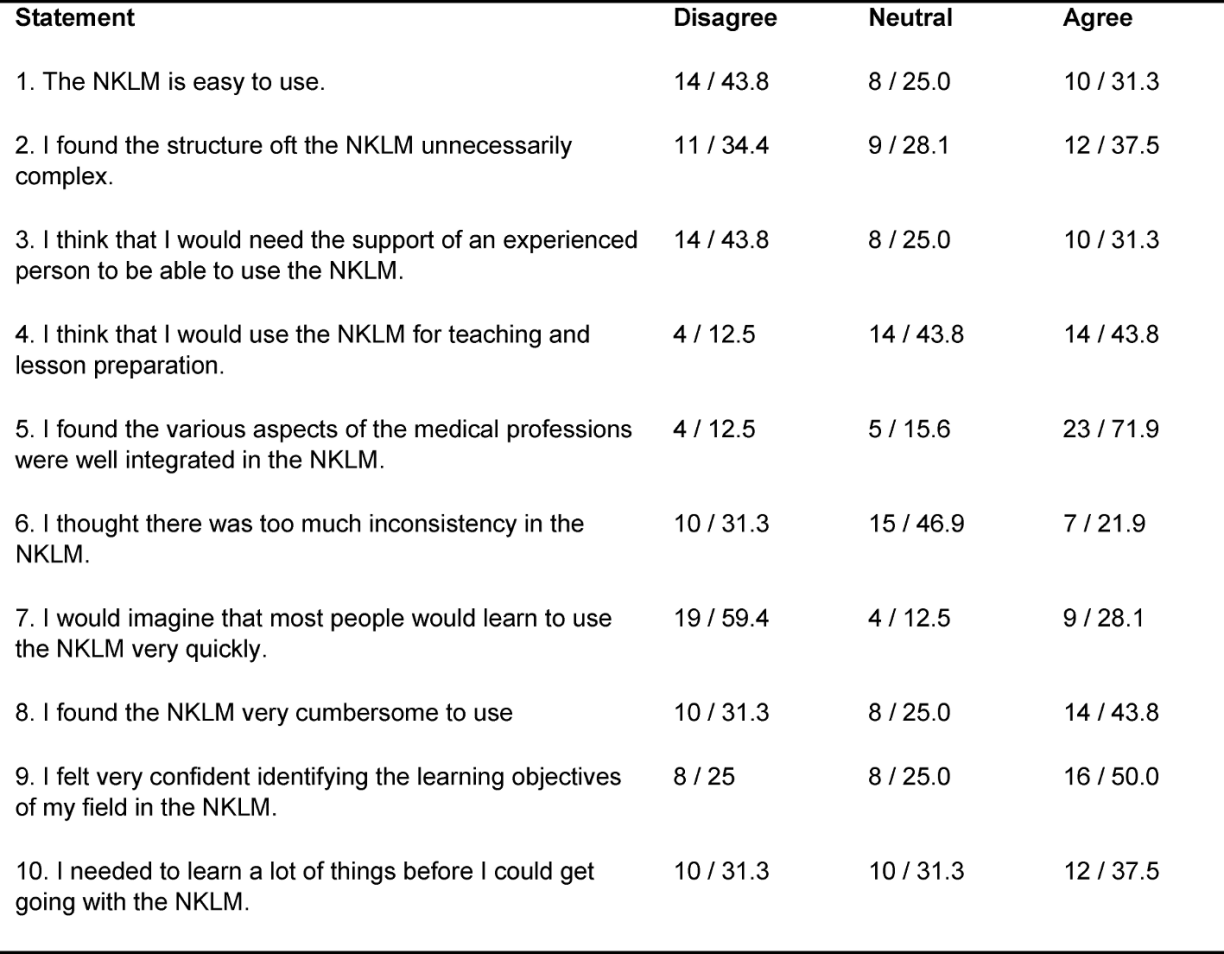
Responses to individual statements in the NKLM System Usability Scale. Distribution of the responses to the single items in total / percent, N=32. The two outer categories on each side of the scale were aggregated e.g., “Strongly Agree and Agree”, into a single value.

**Table 2:**
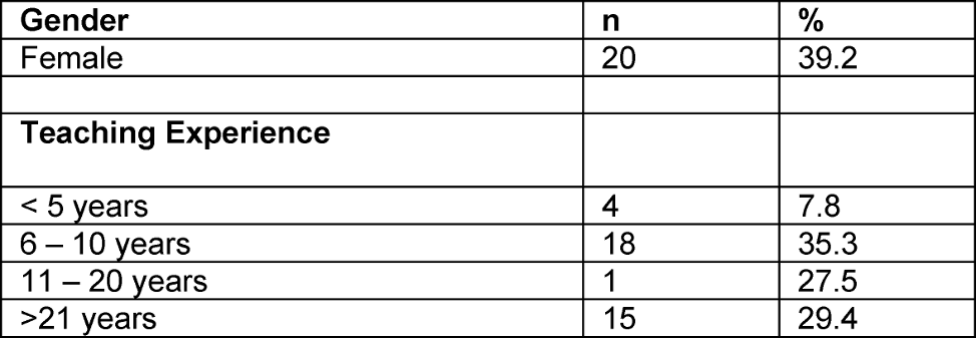
Characteristics of survey participants (n=51 and n=1 not applicable).
